# Evaluating genotype–treatment interactions for high-risk medications in British general practice: a retrospective cohort study using UK Biobank

**DOI:** 10.3399/BJGP.2024.0806

**Published:** 2026-02-01

**Authors:** Kinan Mokbel, Michael Weedon, Rob Daniels, Victoria Moye, Leigh Jackson

**Affiliations:** 1 Department of Health and Care Professions, Faculty of Health and Life Sciences, University of Exeter Medical School, Exeter, UK; 2 Department of Clinical and Biomedical Sciences, Faculty of Health and Life Sciences, University of Exeter Medical School, Exeter, UK

**Keywords:** adverse drug reaction reporting systems, drug-related side effects and adverse reactions, general practice, pharmacogenetics, pharmacovigilance, primary health care

## Abstract

**Background:**

Pharmacogenetics has the potential to optimise drug therapy and reduce adverse drug effects (ADEs) by tailoring treatment to a patient’s genotype, particularly for chronic disorders managed in general practice. However, the adoption of pharmacogenetics in general practice remains slow.

**Aim:**

To evaluate the reproducibility of previously reported associations between genomic variants and medically important adverse drug effects (MIADEs) associated with high-risk medications in general practice.

**Design and setting:**

A retrospective study using data from the UK Biobank (UKBB), a population-based cohort of over 500 000 community-based participants.

**Method:**

High-risk medications prescribed in general practice were identified by linking serious ADEs from the Yellow Card database with English general practice prescription data. These high-risk medications were then cross-examined with genomic variants associated with MIADEs from the Pharmacogenomics Knowledgebase (PharmGKB) to select variant–drug pairs for investigation within the UKBB.

**Results:**

From 78 high-risk medications prescribed in general practice and 56 PharmGKB annotations linked to MIADE risk, *SLCO1B1* rs4149056 was the only variant with guideline-based prescribing recommendations. This variant, along with others of lower evidence levels, was analysed in the UKBB. No genotype–treatment interaction was observed for *SLCO1B1* rs4149056 and statin-related muscle toxicity. Similarly, no interactions were detected for the remaining variants in either secondary or exploratory analyses.

**Conclusion:**

No statistically significant genotype–treatment interactions were observed for MIADE risk associated with high-risk medications in general practice. However, the limited predictive value of the assessed variants may reflect underlying phenotypic imprecision and methodological limitations. Hence, further research is needed to validate these results.

## How this fits in

Pharmacogenetic variants are common in general practice and several high-risk medications have known gene–drug interactions, with *SLCO1B1* rs4149056 currently the only variant supported by guideline-based prescribing recommendations. However, most supporting evidence comes from small studies and lacks validation in large, real-world populations. Using UK Biobank data, this study found no significant genotype–treatment interactions for *SLCO1B1* rs4149056 or other variants in relation to medically important adverse drug effects. These findings suggest that the predictive value of current pharmacogenetic recommendations in general practice remains uncertain and highlights the need for further research to support their clinical application.

## Introduction

All medications carry a risk of causing adverse drug effects (ADEs), which increase morbidity, mortality, and healthcare costs.^1,2^ Currently, the interindividual variability in response to drug treatment is often addressed by adjusting the dose or using a trial-and-error approach, typically after patient harm has occurred.^3^ In the current paradigm of general practice, where most care is managed and medicines are prescribed,^4^ patients and GPs share decision making on which medicines to prescribe. Once this decision has been made, the GP writes the prescription and schedules follow-up appointments with the patient for review. The medication is continued if it is both effective and well tolerated. Conversely, substitution is sought if ADEs occur or if the medication is not effective.^5^ This increases empiricism in the choice of pharmacotherapy in general practice and exposes patients to the possibility of ADEs and ineffective treatments as a result, necessitating a more individualised strategy to reduce such risks.

Individualised treatment approaches could be advanced by using genomic data to identify patients' susceptibility to developing ADEs, complementing rather than substituting current decision making in the prescribing process.^6^ An appropriate setting for the broad adoption of such personalised approaches is general practice, with its well-established infrastructure and the nature of long-term patient care as required for managing chronic conditions, which frequently require multiple medications and ongoing adherence.

Pharmacogenetic variants that modulate drug responses are highly prevalent in the general population,^7–10^ with >60% of patients in primary care prescribed at least one medication with a pharmacogenetic recommendation.^11^ Each of these variants is capable of modulating the effects of multiple medications taken for various conditions. Exposure to medications with pharmacogenetic variants with dosing guidelines in the Pharmacogenomics Knowledgebase (PharmGKB)^12^ was found to be remarkably high, with >80% of patients being exposed to at least one medicine, according to one report.^13^ An analysis of 14 pharmacogenes in the UK Biobank (UKBB) revealed that >99% of participants had a predicted atypical response to at least one medication.^14^

Despite its clear benefits, the integration of pharmacogenetics into clinical practice has been progressing slowly. Although some progress has been made, only a few germline variants have been incorporated into prescription decision-making pathways. Two successful examples include severe hypersensitivity caused by the anti‐HIV/AIDS medication, abacavir,^15^ and severe cutaneous adverse reactions associated with the anticonvulsant medication, carbamazepine.^16^ The limited clinical validity of pharmacogenetic variants remains a major barrier to their integration into prescribing pathways to support decision making in clinical practice.^17^

Overall, the lack of replication is also a widely recognised issue in both pharmacogenetic and genetic-association studies.^18^ Hence, there is a need for significant additional effort to be made in the replication of research, especially in large independent datasets. This is the first study, to the authors' knowledge, in which the accuracy of previously reported associations between variants associated with medically important adverse drug effects (MIADEs) related to high-risk medications in general practice (that is, medicines with high toxicity profiles) has been investigated. A unique aspect of this study is its use of UKBB data, along with the incorporation of real-world general practice prescription records and national pharmacovigilance data for pharmacogenetic investigations.

## Method

### Identification of high-risk medicines in general practice

To identify high-risk medications in general practice, UK pharmacovigilance data on serious and fatal ADEs from the Yellow Card system^19^ were mapped onto English general practice prescription data from OpenPrescribing^20^ for the period January 2016 to January 2021, following previously described methodology.^21^ Briefly, comparative safety charts for clinically significant medicines were generated by analysing safety profiles based on serious and fatal ADE rates per 1 000 000 prescribed items, focusing only on medications initiated or continued in general practice.

Medicines not commonly prescribed in general practice were excluded based on clinical judgement, which corresponded to those with <220 000 prescription items. This approach ensured that the high-risk medicines included in the analysis were those routinely prescribed, clinically relevant to general practice, and sufficiently represented in the dataset. Additional exclusions included medications administered via non-oral routes, highly specialised medicines, and over-the-counter products, as these are typically not initiated or continued in general practice settings. Combination therapies were also excluded to reduce statistical noise and potential bias, as ADE counts for these agents may reflect effects of the individual components as well as the combined formulation, making attribution to the combination therapy unclear. In addition to this focused approach,^21^ which included medications with >220 000 prescribed items, a more comprehensive analysis was conducted of those with >10 000 prescriptions during the same period to ensure broader coverage and enhance the overall comprehensiveness of the study.

To ensure the robustness of this study's list of high-risk medications, three analyses were conducted: a) aggregation (all single and multiple constituent medicines administered through any routes of administration); b) exclusion (excluding multiple constituent medicines and those administered via inappropriate routes); and c) inclusion (medicines administered through routes deemed suitable for general practice). These were applied to both the focused and comprehensive approaches, resulting in six sets of analyses.

Forest plots were generated to illustrate the ADE rates per million items prescribed within each therapeutic class. To identify high-risk medications in general practice for this pharmacogenetic study, high-risk medications were those top-ranked with non-overlapping confidence intervals with other medications in their therapeutic class; those with the narrowest intervals were selected if overlaps occurred. Medicines were excluded if the *P*-value of the *Q*-statistic was <0.05 or if most confidence intervals overlapped.

### Identification of variant–drug pairs associated with MIADEs

Phenotypes and clinical annotations related to ADEs with a high or moderate level of evidence (1A, 1B, 2A, and 2B) in PharmGKB were extracted to identify variants associated with MIADEs.^22^ The primary focus of this analysis was on genetic variants, with PharmGKB Level 1A clinical annotations representing the highest level of evidence. These annotations are supported by variant-specific prescribing recommendations in US Food and Drug Administration (FDA)-approved drug labels or established clinical guidelines.

As a secondary analysis, variants annotated at Levels 1B, 2A, and 2B were examined. Level 1B variants are supported by strong evidence from at least two independent studies but lack formal prescribing guidance. Level 2A and 2B annotations reflect moderate evidence; Level 2A variants are located in Tier 1 Very Important Pharmacogenes (VIPs), indicating greater biological plausibility, while Level 2B variants are not VIPs but meet similar evidence thresholds. The literature mentioned in the clinical annotations was examined as needed and the exclusion and inclusion criteria were applied. Unless their indications overlapped with those of other therapeutic classes, annotations related to anaesthetics and chemotherapy for cancer were excluded. Next, drug–variant pairs that were significantly associated with MIADEs were identified.

The term MIADEs was introduced to address the heterogeneity in the terminology used in the literature to describe the seriousness of ADEs. Although it may appear counterintuitive to introduce another term here, the purpose of this introduction is to unify the existing disparate classifications used in the literature to characterise the seriousness of ADEs and enhance the consistency of ADE reporting, thus facilitating more transparent communication and allowing more robust comparability across different studies. MIADEs address the need for a comprehensive term that captures events classified as either serious or severe by investigators, meet the World Health Organization criteria for seriousness,^23,24^ classified as severe,^25^ or recognised as designated or important medical events.^26,27^ Composite toxicity outcomes were included if at least one incorporated endpoint satisfied the MIADE criteria, but underspecified toxicity outcomes and unspecified treatment discontinuation were excluded.

Haplotypes and star alleles were interpreted using relevant allele nomenclature to create more precise and interrogable genotypes.

### UK Biobank analyses

#### Description of the study population

The UKBB is a population-based cohort comprising >500 000 community-based participants who attended one of the evaluation centres in Wales, Scotland, or England between 2006 and 2010.^28^ Blood samples were collected for genomic and biomarker analyses along with health-related data from the baseline assessment. The participants were followed up after the baseline assessment and their health records data were regularly updated. This included self-reported non-cancer illness data collected at baseline and during follow-up available up to February 2022, as well as hospital inpatient records (Hospital Episode Statistics [HES]) coded using the International Classification of Diseases, 9th Revision (ICD-9) and 10th Revision (ICD-10), with data available until February 2022 for England and Scotland, and February 2018 for Wales. Linkages to national death registries (England, Scotland, and Wales until February 2022) and cancer registries (to May 2022 for Scotland and February 2022 for England and Wales) were also available. These data allowed comprehensive longitudinal ascertainment of MIADEs following treatment initiation.

Patients who self-reported taking relevant medications at baseline were identified using UKBB field ID 20003, which captures self-reported medication use at the time of enrolment during baseline assessment interviews, which began in January 2012. The specific medication codes included in the analysis are detailed in Supplementary Box S1.

As the samples available for other ancestral groups in the UKBB were insufficient in size to draw any reliable conclusions or robust subgroup analyses, the analyses were limited to 389 805 unrelated individuals with genetically determined European ancestry and included principal components of ancestry in the regression analyses to account for population stratification bias.

#### Ascertainment of biomarkers and other phenotypes

The study used data from baseline measurements, updated HES, and follow-up visits. To generate phenotypes, the ICD-9, ICD-10, and self-reported codes were used. Details of the UKBB field IDs and codes used to ascertain baseline measurements and incident MIADE phenotypes, defined as events occurring after treatment initiation between baseline and follow-up, are provided in Supplementary Boxes S2 and S3. The ICD-9 and ICD-10 codes were categorised to represent phenotypes and diseases with underlying biological relevance. The complete list of ICD-9 and ICD-10 codes can be found on the UKBB data showcase.^29,30^

A body mass index (BMI) ≥41.66, or three standard deviations above the average BMI of unrelated Europeans in the UKBB, was used to classify weight gain as severe. A non-fasting triglyceride level >2.3 mmol/L was considered to indicate hypertriglyceridaemia.^31^

#### Variant selection and genotyping procedures

High-risk medications in general practice and variants conferring a risk of MIADEs were cross-examined to identify variants that confer a risk of MIADEs related to the high-risk medications in general practice to be interrogated in the UKBB cohort. All the medications in a therapeutic class were included in the UKBB analyses when a clinical annotation in PharmGKB was associated with the entire therapeutic class. The single nucleotide polymorphism genotyping array data used in this study were generated by the UKBB and subjected to rigorous quality control.^32^

### Statistical analysis

The statistical methods for analysing safety data have been previously described.^21^ Regression analyses were employed to investigate associations between genetic variants and MIADEs, utilising both main effects and interaction models. The statistical analyses were adjusted for assessment centres, the first five genetic principal components of ancestry and potential confounders as per the PharmGKB annotations, or the original research articles.

To correct for multiple testing in the secondary analysis, a Bonferroni correction was applied, yielding a significance threshold of *P*-value <0.004 (0.05/13 tests). In exploratory analyses, where individual medications were assessed separately or definitions of toxicity phenotypes were relaxed, a stricter Bonferroni-corrected threshold of *P*-value <0.001 (0.05/130 tests) was applied. Stata/SE (version 16.0) was utilised for data management and statistical analysis, and the analyses were two-tailed. The 'regress' command for linear regression and 'logistic' for logistic regression models were employed.

## Results

### High-risk medicines in general practice were identified

**Figure 1.**
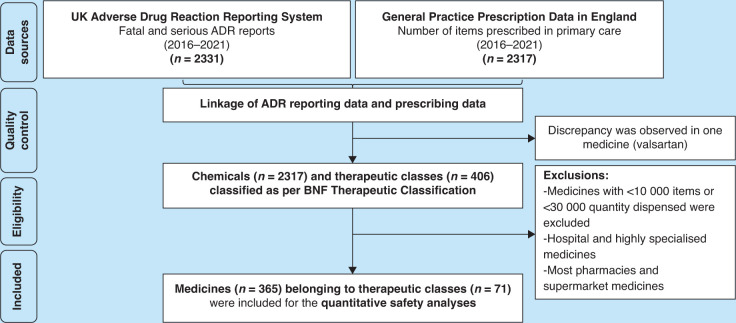
The process of mapping data on adverse drug effects (ADEs) onto general practice prescription data using the comprehensive approach. A flow chart demonstrates the method utilised to identify high-risk medicines. Data on serious and fatal ADEs from the Yellow Card database was linked to general practice prescription data in England. Exclusion criteria were further applied to identify top-ranked medicines (that is, high-risk medicines). ADR = adverse drug reaction. BNF = British National Formulary.

By mapping UK pharmacovigilance data onto prescribing data, the authors’ previously published approach identified 137 clinically significant medications across 26 therapeutic classes in the focused analysis,^21^ and 365 medications across 71 therapeutic classes in the comprehensive analysis (Figure 1). Supplementary Figure S1 displays the quantitative comparative safety forest plots for both the focused and comprehensive analyses, incorporating aggregation, exclusion, and inclusion criteria. By examining confidence intervals across six sets of forest plots, 78 high-risk medications were identified, with the top-ranked ones detailed in Supplementary Box S4.

### **Variant–drug pairs previously identified as being associated with** MIADEs

**Figure 2.**
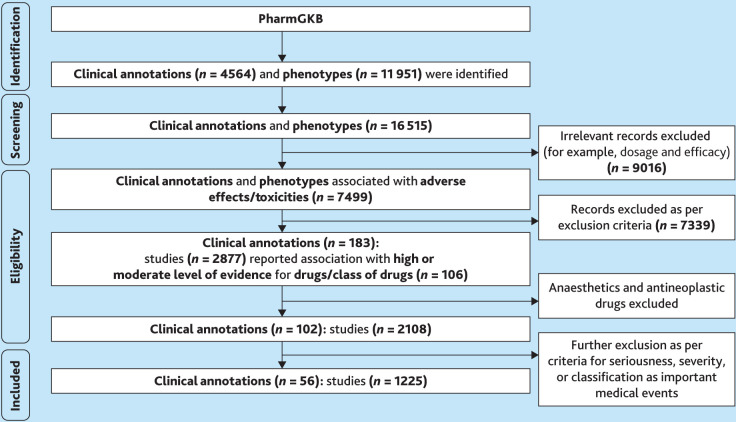
A flow chart illustrating the identification procedure of variant–drug pairs for MIADEs in PharmGKB. Several clinical annotations included >1 toxicity outcome and were therefore split into separate records during curation. For this reason, the number of clinical annotations at this stage does not correspond to a direct subtraction from the previous step.MIADE = medically important adverse drug effect. PharmGKB = Pharmacogenomics Knowledge Base.

The study identified 102 PharmGKB clinical annotations associated with ADE risk at high or moderate evidence levels (1A, 1B, 2A, and 2B), of which 56 pertained specifically to MIADEs (Figure 2). The complete list of drug–variant pairs associated with MIADEs can be found in Supplementary Box S5.

### Cross-examination of high-risk medicines and variant–drug pairs

After cross-examining 56 clinical annotations related to the risk of MIADEs at high or moderate evidence levels (1A, 1B, 2A, and 2B) and 78 high-risk medications in general practice, three therapeutic classes to be investigated in the UKBB population were identified: statins, non-steroidal anti-inflammatory drugs (NSAIDs), and antipsychotics.

**Figure 3.**
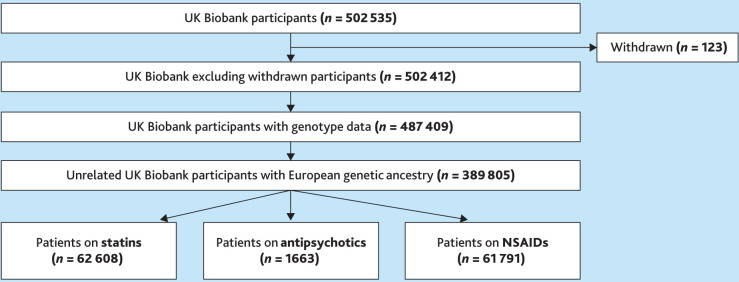
UK Biobank participants with European genetic ancestry were eligible for the secondary analysis. A cohort flow chart of the UK Biobank participants included in the secondary analysis with adequate treatment and genomic data. NSAID = non-steroidal anti-inflammatory drug.

Among these, only one variant, rs4149056 in *SLCO1B1*, was annotated at Level 1A. Figure 3 displays the cohort flow chart of UKBB participants included in the secondary analysis based on eligibility and availability of adequate treatment and genomic data.

The key characteristics of the UKBB participants are summarised in Table 1. A summary of the variants examined and their frequency in UKBB participants is provided in Table 2. All the variants extracted from the imputed data were imputed with a high degree of confidence (>99.7%).

**Table 1.** Comparison of key characteristics and summary statistics for participants on the three investigated treatments versus the rest of the UK Biobank participants

Main characteristics and toxicity measures	Participants on investigated treatments, *n* ^a^	Rest of UKBB participants, *n* ^a^
**Statins**	62 608	439 928
Age, years, mean (SD)	62 (6.0)	56.3 (8.0)
BMI, kg/m^2^, mean (SD)	29.4 (5.0)	27 (4.6)
Myopathy, rhabdomyolysis, and muscular diseases	408	1819
**Antipsychotics (group 1)^b^**	1125	501 411
Age, years, mean (SD)	54.6 (8.0)	57.2 (8.0)
BMI, kg/m^2^, mean (SD)	29.6 (6.1)	27.4 (4.8)
Hypertriglyceridaemia (high versus low)	478	209 966
Severe weight gain (BMI ≥41.66)	57	118 826
**Antipsychotics (group 2)^c^**	1663	500 873
Age, years, mean (SD)	55.7 (8.0)	57.2 (8.0)
BMI, kg/m^2^, mean (SD)	29.4 (6.0)	27.4 (4.8)
Hyperprolactinaemia	6	35
Tardive dyskinesia	2	24
Severe weight gain (BMI ≥41.66)	78	118 805
**NSAIDs**	61 791	440 745
Age, years, mean (SD)	55.9 (8.1)	57.5 (8.0)
BMI, kg/m^2^, mean (SD)	28.1 (5.1)	27.2 (4.7)
Gastrointestinal bleeding	2398	16 672

^a^Unless otherwise stated. ^b^Amisulpride, aripiprazole, clozapine, haloperidol, olanzapine, paliperidone, quetiapine, risperidone, and ziprasidone (based on PharmGKB clinical annotations; see Supplementary Table S1). ^c^All antipsychotics included in the UK Biobank medication codes for eligibility (see Supplementary Box S1). Group 1 is a subset of group 2 and the two groups are not mutually exclusive, which is why their counts do not sum to the total number of unique antipsychotic users (*n* = 1663) shown in Figure 3. BMI = body mass index. NSAID = non-steriodal anti-inflammatory drug. SD = standard deviation. UKBB = UK Biobank.

**Table 2.** The genomic variants included in the secondary analysis and their prevalence in the UKBB

Gene	Variant ID	Directly genotyped or imputed	Imputation score *R* ^2a^	Chromosome number	Position	Allele 1	Allele 2	Minor allele UKBB	MAF UKBB (unrelated Europeans)
*NAT2*	rs1041983	Imputed	0.999	chr8	18 257 795	C	T	T	0.33
*NAT2*	rs1799930	Genotyped	N/A	chr8	18 258 103	G	A	A	0.30
*DPP6*	rs6977820	Genotyped	N/A	chr7	154 072 020	T	C	T	0.28
*COQ2*	rs4693075	Genotyped	N/A	chr4	84 192 168	G	C	G	0.38
*MC4R*	rs489693	Genotyped	N/A	chr18	57 882 787	C	A	A	0.33
*GATM*	rs1346268	Imputed	1	chr15	45 673 029	T	C	C	0.26
*GATM*	rs1719247	Imputed	0.995	chr15	45 620 985	C	T	T	0.26
*SLCO1B1*	rs4149056	Genotyped	N/A	chr12	21 331 549	T	C	C	0.15
*ANKK1*	rs1800497	Genotyped	N/A	chr11	113 270 828	G	A	A	0.21
*CYP2C9*	rs1057910	Genotyped	N/A	chr10	96 741 053	A	C	C	0.06

a *R* ^2^ is the squared correlation between input genotypes and imputed dosages (that is, true and inferred genotypes). MAF = minor allele frequency. N/A = not applicable (imputation score applies only to imputed variants). UKBB = UK Biobank.

### Evaluation of previously reported associations in the UK Biobank cohort

Overall, eight variants were investigated in the UKBB analysis:

- four in *SLCO1B1, GATM*, and *COQ2* genes were associated with statin-induced muscle toxicity;
- one in *CYP2C9* with an elevated risk of acute gastrointestinal bleeding caused by NSAIDs; and
- three in *MC4R, DPP6*, and *ANKK1* with the risk of tardive dyskinesia, hyperprolactinaemia, hypertriglyceridaemia, or severe weight gain related to antipsychotics.

*SLCO1B1* rs4149056, the only variant with a Level 1A clinical annotation and previously associated with statin-induced muscle toxicity, showed no evidence of a genotype–treatment interaction in this cohort, even in exploratory analyses. Furthermore, the current study was unable to replicate any previously reported associations between the other genetic variants and the risk of MIADEs, including in exploratory analyses (Table 3). Although two associations were statistically significant in the main effects model, none met the stringent significance threshold in the interaction model. The complete results of the analysis, including the statistical models for the main effects and interaction terms, are shown in Supplementary Table S1.

**Table 3.** Summary of secondary analysis findings and PharmGKB evidence level updates

Treatment(s) and adverse drug effect variant	Genetic model	UKBB study^a^	Level of evidence (PharmGKB)	
		OR (95% CI), *P*-value	July 2020	November 2024
**Statins**				
Myopathy, muscular diseases				
rs4149056 [C]	Dominant CC + CT versus TT	1.03 (0.80 to 1.32), 8.31E-01	1A, 2A	1A, 2A
rs1719247 [C]	Recessive CC versus CT or TT	1.12 (0.89 to 1.40), 3.38E-01	2B	3
rs1346268 [T]	Recessive TT versus CC or CT	1.14 (0.91 to 1.43), 2.57E-01	2B	3
	Dominant CT + TT versus CC	0.92 (0.60 to 1.41), 6.91E-01	2B	3
rs4693075 [G]	Dominant GG or CG versus CC	1.02 (0.81 to 1.28), 8.90E-01	2B	3
**Antipsychotics (group 1)**				
Hypertriglyceridaemia				
rs489693 [A]	Recessive AA versus AC or CC	1.17 (0.78 to 1.77), 4.52E-01	2B	3
Severe weight gain				
rs489693 [A]	Recessive AA versus AC or CC	1.59 (0.78 to 3.26), 2.03E-01	2B	3
rs1800497 [A]	Dominant AA + AG versus GG	1.12 (0.65 to 1.93), 6.92E-01	2B	Annotation removed
**Antipsychotics (group 2)**				
Hyperprolactinaemia				
rs1800497 [A]	Dominant AA + AG versus GG	9.04 (0.91 to 89.96), 6.03E-02	2B	3
Severe weight gain				
rs489693 [A]	Recessive AA versus AC or CC	1.53 (0.82 to 2.86), 1.79E-01	2B	3
rs1800497 [A]	Dominant AA + AG versus GG	1.12 (0.70 to 1.78), 6.45E-01	2B	Annotation removed
Tardive dyskinesia				
rs1800497 [G]	Additive G	0.61 (0.29 to 1.27),^b^ 1.85E-01	2B	Annotation removed
rs6977820 [T]	Dominant CT + TT versus CC	1.01 (0.39 to 2.62),^b^ 9.83E-01	2B	3
**NSAIDs**				
Acute gastrointestinal bleeding				
rs1057910 [C]	Additive C	0.99 (0.87 to 1.13), 9.06E-01	2A	3

ab Interaction terms model unless annotated otherwise (for example, main effects). Main effects. Group 1 antipsychotics: amisulpride, aripiprazole, clozapine, haloperidol, olanzapine, paliperidone, quetiapine, risperidone, ziprasidone (based on PharmGKB clinical annotations; see Supplementary Table S1). Group 2 antipsychotics: all antipsychotics included in the UK Biobank medication codes for eligibility (see Supplementary Box S1). NSAID = non-steriodal anti-inflammatory drug. OR = odds ratio. PharmGKB = Pharmacogenomics Knowledge Base. UKBB = UK Biobank.

## Discussion

### Summary

Patients and GPs are optimistic regarding the potential of using genomic variants that confer a risk of ADEs to optimise prescribing practices and improve safety.^11^ Using UKBB data, this extensive analysis aimed to validate the reproducibility of previously reported associations between genomic variants and MIADEs related to high-risk medications in general practice. *SLCO1B1* rs4149056, the only Level 1A variant, showed no genotype–treatment interaction. No other variant–MIADE associations were replicated, even in exploratory analyses, contributing to the ongoing discussion about the clinical utility and generalisability of pharmacogenetics in primary care.^33–36^ However, it is possible that the failure to replicate was influenced by differences in population structure, phenotyping definitions, or underpowered subgroup analyses for rare variants or the frequency of MIADEs.

### Strengths and limitations

The two main strengths of the current study are the large sample size provided by the UKBB and the detailed documentation of relevant phenotypes with longitudinal data, including HES and follow-up data, which are substantially longer than those used in the initial studies. Compared with the UKBB, the majority of the initial studies were smaller in size and had shorter follow-up periods, which can limit the observation of certain MIADEs. The large sample size of the UKBB cohort enabled the detection of relatively uncommon MIADEs, particularly among individuals who had variants with minor allele frequencies <5%.

Although this study offers valuable insights, a few limitations warrant consideration. First, the identification of high-risk medications involved mapping data from the Yellow Card database with the English prescription database, which warrants caution because of the inherent limitations in both databases.^21^ However, these limitations are expected to affect all medications equally, meaning that the relative rankings reflecting ADE rates per unit prescribed are unlikely to be affected.

Second, the phenotypic data within the UKBB, particularly those self-reported by participants at baseline, might vary in reliability and quality, hindering the accurate identification of those with the relevant conditions.^37^

Third, since this analysis involved UKBB participants with European ancestry, the generalisability of the findings to populations from other ethnic groups is limited.

Fourth, participants in the UKBB cohort with risk variants may be generally healthier than carriers in the general population, which may attenuate potential pharmacogenetic effects.^38^ Nevertheless, the current study’s results are less confounded than those from conventional clinical trials as UKBB participants were not informed whether they carried a particular genetic variant.

Finally, it is also possible that some true genotype–treatment interactions may have gone undetected because of the stringent multiple testing correction applied given the extensive and thorough scope of the study's analysis, where several genotype–drug interactions were tested across various toxicities. Although controlling for multiple tests is essential to minimise type I errors, it also increases the risk of type II errors (missing true positive effects), particularly in large datasets such as the UKBB dataset, where potential noise can further obscure true associations. Thus, smaller and more targeted investigations might detect significant findings for specific genotype–drug toxicity combinations that the current study was not sufficiently powered to identify. For example, future research may benefit from focusing solely on Level 1A variants to ensure the most robust and actionable findings for clinical implementation, thereby aligning with current Clinical Pharmacogenetics Implementation Consortium (CPIC) standards for recommended pharmacogenetic testing.

### Comparison with existing literature

The PREPARE study recently implemented pre-emptive pharmacogenetic testing to evaluate its potential to prevent ADEs.^39^ The study, which involved 6944 patients, reported that genotype-guided treatment resulted in a 30% reduction in clinically relevant ADEs. However, the PREPARE study has been subject to criticism because of loss of approximately 10% of patients at follow-up, which disproportionately had an impact on the intervention group.^40^ Notably, the authors did not report how the unblinded trial design affected ADE reporting.^41^ Furthermore, even among those who did not receive actionable results, there was a reduction in ADEs, which may indicate that people were reluctant to report ADEs after pharmacogenetic testing.^42^ Moreover, concerns regarding the heterogeneous effects across various EU member states raises further questions about the feasibility of broadly implementing pharmacogenetic testing. Other concerns also include small patient groups, heterogeneity of medications included, and the aggregate analysis of all medications. There are also reservations and doubts about the actionability assigned by the Dutch Pharmacogenetics Working Group guidelines to the drug–variant combinations used.^43^

In evaluating the clinical validity of pharmacogenetic variants, the authors of the current study recognise that only one of the eight variants in this study achieved a PharmGKB Level 1A evidence rating, which denotes high clinical relevance and is supported by prescribing guidelines or FDA-approved label annotations. Owing to their strong evidentiary support, Level 1A variants are the only ones presently recommended for clinical use. By including variants with lower evidence ratings (1B, 2A, and 2B), the current study aimed to explore the broader spectrum of pharmacogenetic markers linked to MIADEs in general practice, although the authors acknowledge that these are not currently supported by prescribing guidelines and therefore lack direct clinical utility.

In this study, the authors curated variant evidence levels from PharmGKB at the outset of the research, aiming to capture the most relevant clinical data available at the study’s inception. When this study was being written in 2024, PharmGKB reassessed and downgraded the evidence levels for the majority of these variants to Level 3, except for *SLCO1B1* rs4149056 and statin-induced muscle toxicity, denoting a limited level of evidence supporting clinical associations. Notably, PharmGKB has re-evaluated and removed the clinical annotation for the *ANKK1* rs1800497 variant regarding antipsychotic-induced severe weight gain and tardive dyskinesia. This decision reflects the current understanding that the evidence supporting these associations is insufficient for clinical application (see Table 3). Thus, the findings in the current study align with the revised classification as no significant genotype–treatment interactions were found, indicating a limited predictive value of these variants for MIADEs. This alignment with updated evidence highlights the robustness of the current study's approach and reflects the study’s relevance to current clinical standards. Therefore, the authors recommend that future pharmacogenetic studies integrate the latest evidence as it becomes available, as such updates reflect the iterative nature of scientific progress in this ever-evolving field and enhance the robustness and clinical relevance of findings.

According to the PharmGKB evidence rating, only the *SLCO1B1* variant rs4149056, which is associated with statin-induced muscle toxicity, currently holds a Level 1A evidence rating. However, the PharmGKB’s current designation of a high evidence level for the association between rs4149056 and statin-related musculoskeletal toxicity is still questionable,^43^ especially in light of the multiple studies that have failed to find a significant association.^44–55^ The majority of studies cited by PharmGKB to support the high level of evidence assigned to this association investigated the associations between the rs4149056 genotype and statin plasma concentrations or the pharmacokinetic variability of those drugs.^44,56–92^ However, a sizable portion of these pharmacokinetic studies either failed to confirm the significance of this association^44,66–69,76,93^ or produced findings that contradict those previously reported.^64,87^ It is worth highlighting that the majority of patients who start statin therapy eventually discontinue it, mostly because of ADEs. As a result, over half of the potential benefits of statins are still not realised.^94^ Therefore, other personalised strategies are needed to shift such unpredictable ADEs into predictable occurrences. In addition to decreasing the frequency and severity of related ADEs, this approach can help improve the efficacy of statins.

Although statins share a common mechanism as 3-hydroxy-3-methylglutaryl coenzyme A (HMG-CoA) reductase inhibitors and are often used interchangeably in general practice,^95^ the current study’s analysis acknowledges the critical role of variant-specific and drug-specific guidelines in the pharmacogenetics of statins. According to the CPIC, the impact of rs4149056 on statin metabolism is not uniform across all statins, suggesting a differential genetic impact across statins.^96^ This evidence underpins the CPIC recommendation to consider individualised statin selection and dosing on the basis of the *SLCO1B1* genotype to reduce ADEs, such as myopathy. The current study endeavoured to replicate associations as reported in the literature that formed the basis for PharmGKB’s evidence levels. These studies generally analysed statin treatment as a single class, commonly grouping statins together rather than evaluating the effects of individual statins separately.^97,98^

In addition to this class-wide analysis, subgroup analyses were conducted to assess the interaction between rs4149056 and muscle toxicity in UKBB participants on individual statins. This approach aligns more closely with CPIC guidelines to ensure clinically relevant findings and accommodates the differential pharmacokinetic variations across individual statins. Analysing these pharmacogenetic associations is more relevant to general practice; however, no significant genotype–treatment interactions were detected, even without adjustments for multiple testing (see Supplementary Table S1). Moreover, although grouping certain medications within therapeutic classes in the quantitative safety analyses may prompt questions owing to pharmacological and pharmacogenetic differences within each class, these classifications are in accordance with those in the General Practice Prescribing Database, where medications are organised according to their positions in the British National Formulary. In the current study, these groupings were retained to enable broader applications of these safety charts beyond pharmacogenetics, such as offering general practices and patients real-world drug safety data to support informed decision making.

### Implications for research and practice

The study's findings indicate that the analysed variants may have limited predictive value for MIADEs within this cohort. However, this does not preclude their potential relevance in other clinical settings or populations, particularly where variant frequencies, prescribing patterns, phenotype definitions, or ascertainment differ. These results should therefore be interpreted in light of the study’s design, population characteristics, and methodological limitations. Yet, the current results provide valuable insights for policymakers and stakeholders considering the implementation of pharmacogenetic testing in general practice. This study also underscores the necessity of integrating the most relevant pharmacogenetic recommendations to enhance precision in medication management.

These findings are particularly relevant in light of the PROGRESS study,^99^ which is evaluating the feasibility of implementing pre-emptive pharmacogenetic panel testing in primary care to guide prescribing for commonly used drugs such as statins, selective serotonin reuptake inhibitors, tricyclic antidepressants, or proton pump inhibitors. Although the current study found limited evidence of genotype–treatment interactions at the population level, studies like PROGRESS highlight the value of real-world implementation research in assessing clinical utility and informing future integration of pharmacogenetics into routine care.

In conclusion, this investigation did not identify statistically significant evidence to support previously reported associations between pharmacogenetic variants and MIADEs linked to high-risk medications in general practice. However, the findings should be interpreted in light of methodological and data limitations, reinforcing the need for cautious interpretation and further targeted research in larger and more diverse populations. Future studies with more refined phenotypes, ancestry-diverse cohorts, and clinical trial-level outcome validation may yield clearer evidence regarding the predictive value of these pharmacogenetic markers.

## Data

Data related to the Yellow Card system, English general practice prescriptions, and Pharmacogenomics Knowledgebase, as discussed in this study, are freely available and can be accessed on their respective websites by those interested in using them for non-commercial purposes. The genetic and phenotypic data from the UKBB can be accessed by applying through their website (https://www.ukbiobank.ac.uk/use-our-data/apply-for-access). The authors cannot directly grant access to the specific data fields used in this study. All other data relevant to this study are included in the article.
